# Mouse Gnal transcripts and transcriptomics in isolated dystonia

**DOI:** 10.21203/rs.3.rs-7222154/v1

**Published:** 2025-08-29

**Authors:** Ajeet Kumar, Samira Saeirad, Mark S. LeDoux

**Affiliations:** University of Memphis; University of Memphis; University of Memphis

**Keywords:** Dystonia, Gnal, RNA-se0071, Striatum, Cerebellum

## Abstract

Heterozygous loss-of-function *GNAL* mutations are one established cause of isolated dystonia and hyposmia. Homozygous *GNAL* mutations have been reported in siblings with generalized dystonia and intellectual disability. *GNAL* encodes major [NM_001369387.1; Gα(olf)] and long [NM_182978.4; XLGα(olf)] isoforms. In striatal medium spiny neurons, dopamine D1 receptors and adenosine A2a receptors are coupled to adenylyl-cyclase through a heterotrimeric G-protein complex composed of Gα(olf), Gβ2, and Gγ7 subunits. In the cerebellum, Gα(olf) colocalizes with cell-surface corticotropin-releasing factor receptors (CRF-RI/II) which take part in climbing fiber signaling. In contrast, XLGα(olf) may take part in cell-cycle control and development. *In situ* hybridization (ISH) showed that XLGα(olf) mRNA was more broadly distributed in mouse brain than Gα(olf) mRNA. In the cerebellum, XLGα(olf) mRNA was seen in all layers of cerebellar cortex while Gα(olf) mRNA was mainly limited to Purkinje cells. Gα(olf) showed higher expression than XLGα(olf) in the olfactory bulb and striatum, and lower expression than XLGα(olf) in cerebral cortex, cerebellar cortex, and hippocampus. Dysregulated genes identified in *Gnal*^+/−^ mouse brain contribute to signaling (*Slc5a7, Cbln2, Glra3, Rtn4rl2*), anatomical structure development including dendritogenesis (*Slc5a7, Cbln2, Glra3, Rtn4rl2, XLr3b, Mmp12, Rtn4rl2, Cd74, Kirrel2*), and DNA-templated transcription (*Lhx9, Basp1, Mmp12, Cd74*). Analyses of ClinVar and gnomAD databases suggest that highly deleterious *GNAL* variants isolated to Exon 1 of the long isoform are less likely to be pathogenic than those isolated to Exon 1 of the major isoform. This work forms a platform for continued study of Gα(olf) and XLGα(olf) in dystonia, hyposmia, and intellectual disability.

## Introduction

Dystonia, a genetically and clinically heterogeneous hyperkinetic movement disorder, is distinguished by abnormal, often repetitive movements and postures, that can occur spontaneously or be triggered by voluntary activity^1,2^. Monogenic forms of dystonia are categorized as isolated, combined, or complex^2,3^. *GNAL* mutations (DYT25) are one cause of isolated dystonia^4,5^. *GNAL*, located on chromosome 18p11, encodes a heterotrimeric G-protein α subunit G(olf) of the G protein receptor, which causes cAMP generation and activation of the protein kinase A signaling cascade^6–10^. In striatal medium spiny neurons (MSNs), Gα(olf) associates with Gβ2γ7 subunits to form a heterotrimer^6^. Notably, the Gα(olf)β2γ7 heterotrimer functions to transmit signals from D1 dopamine receptors in the striatal direct pathway (dMSN) and adenosine 2A receptors (A2AR) in the striatal indirect pathway (iMSN)^11^. Accordingly, *GNAL* loss-of-function leads to impaired signal transduction of the D1R- and A2AR-mediated striatal dopamine pathways^12,13^.

*GNAL* also encodes a long isoform [NM_182978.4; XLGα(olf)]. XLGα(olf) has shown Golgi and perinuclear expression in transfected Cos7 cells^14^ and antibodies targeting XLGα(olf)/Gα(olf) have shown evidence for both nuclear and cytoplasmic localization (The Human Protein Atlas, www.proteinatlas.org)^5^. XLGα(olf) harbors a nuclear localization signal. XLGα(olf) regulates expression of CDKNA1B (cyclin-dependent kinase inhibitor 1B [p27^Kip1^]) in a COPS5 (COP9 signalosome subunit 5 variant) and CKD2 dependent manner^15^. These interactions point to a direct role for XLGα(olf) in DNA damage repair and cell-cycle control^16^.

Immunohistochemistry with a rabbit polyclonal antibody was used to localize Gα(olf)/XLGα(olf) immunoreactivity (IR) to the olfactory bulb, striatum, thalamus, cerebral cortex, substantia nigra, and cerebellum in rat brain^5^. The neural distribution of mouse *Gnal* transcripts was explored using RNAscope^™^ probes Mm-*Gnal*-O1 (NM_177137.5, target region bp 426–1462) and Mm-*Gnal*-C2 (NM_010307.2, target region bp 137–1081)^17^. These probes were not isoform specific. *Gnal* transcripts were localized to cerebellar Purkinje cells, iMSNs, dMSNs, striatal cholinergic interneurons, cerebral cortex, and the substantia nigra.

The goals of the proposed experiments were to examine the expression and localization of the long and major isoforms of *Gnal* in mouse brain, effects of Gα(olf)/XLGα(olf) haploinsufficiency on striatal and cerebellar transcriptomes, and relative deleteriousness/pathogenicity of *GNAL* variants isolated to the long and major isoforms. This work forms a framework for future more focused studies on the neurobiology and pathobiology of Gα(olf) and XLGα(olf), and informs clinical-genetic assessment of *GNAL* variants.

## Methods

### Animals

Our experimental protocols strictly adhered to the Guide for the Care and Use of Laboratory Animals by the National Academy of Sciences. Additionally, the University of Memphis Animal Care and Use Committee of the University of Memphis approved all procedures. *Gnal*^+/−^ mice were rederived from sperm stored at Virginia Commonwealth University and maintained on a C57BL/6J background^18^. Mice were kept at constant temperature and humidity on a 12-hour day/night cycle with ad libitum access to water and food. All methods are reported in accordance with ARRIVE guidelines.

### Real-time quantitative polymerase chain reaction (RT-qPCR) analysis

Four wild-type 14-week-old C57BL/6J mice (two male and two female) were euthanized with pentobarbital. Brains were rapidly removed from the cranial cavity, rinsed with saline, and sectioned into sharply dissected blocks of tissues (olfactory bulb [OB], frontal cerebral cortex, striatum, hippocampus, and cerebellum). Total RNA was isolated from the dissected tissues TRIzol^®^ reagent (#15596026, Ambion by Life Technologies, Carlsbad, CA, USA). In an E-tube, tissue was mixed with 500 μL of TRIzol reagent and rigorously mixed until it turned cloudy and stored in −80°C overnight. The next day, we thawed the mixture and centrifuged it at 10,000 rpm at 4°C to yield a clear solution devoid of cellular debris. We added 200 μL of chloroform and vortexed rigorously for 3 min at RT and kept on ice for 5 min. Further, the solution containing E-tubes was centrifuged at 13,000 rpm for 10 min at 4°C, and the top layer was collected as RNA mix in a separate E-tube, and absolute isopropanol was added, and the solution was stored at −20°C for 20 min. The E-tube was centrifuged at 13,000 rpm for 15 min at 4°C to get the total RNA pellet on the bottom. The pellet was washed and centrifuged at 13,000 rpm with 70% ethanol 2X, and the pellet was dried for 5 min at RT before adding DEPC-DW (20 μL) in an E-tube. Optical density (OD) data were collected to assess purity and calibrate the total RNA concentration (1μg) before cDNA synthesis. 5X PrimeScript^™^ RT Master Mix (Perfect Real Time, #RR036A, Takara Bio Inc., Japan) was used to generate cDNA. A reverse transcription reaction was performed for 15 min at 37°C, 5 s at 85°C, and then stored at 4°C after gently mixing the solution. Again, OD was collected to calibrate the concentration of the cDNA (100 ng). GoTaq qPCR Mix (#A6001, Promega Corporation, Madison, WI, USA) was used to perform SYBR-green-based qPCR experiments, and the PCR composition and thermal cycler protocol were followed as instructed by the manufacturer in triplicate (384-well plate) step 1; at 95°C for 3 min, step 2; denaturation 95°C for 10 s, annealing at 55°C for 30 s, and amplification at 55°C for 30 s (39 cycles) and final step 3; at 95°C for 1 min with QuantStudio^™^ 6 Flex System (Thermo Fischer Scientific, Waltham, MA, USA). The results show the curve and CT values of major *Gα(olf)* and long *XLGα(olf)* transcripts with *β-actin*. The ΔΔCT values were quantified and normalized to the *β-actin*. GraphPad was used to generate the column plot. The following forward (FP) and reverse (RP) primers were used for RT-qPCR:

*β-actin* [FP {GGCTGTATTCCCCTCCATCG}, RP {CCAGTTGGTAACATGCCATGT}],

major isoform [FP {ACGACCTGCCTTTAAGGAGC}, RP {CGGTCAGGCAAGTAGGAAGG}], and long isoform [FP {AAGGATGCACAATGGCCCTT}, RP {CTGTAGCATAGGCCCATCGG}].

### RNAscope single-molecule fluorescence in situ hybridization (smFISH)

In collaboration with Advanced Cell Diagnostics (acdbio.com; Newark, CA, USA), we designed mouse *Gnal* probe set Mm-*Gnal*-tv1 targeting base pairs 2–411 of NM_010307.3 which encodes Gα(olf) and probe set Mm-*Gnal*-tv2 targeting base pairs 2–579 of NM_177137.5 which encodes XLGα(olf). Mm-*Gnal*-tv2 probes do not detect NM_010307.3. Mm-Gnal-tv1 probes do not detect coding regions of NM_177137.5. Adult (N = 4, 3 months of age) C57BL/6J mice were deeply anesthetized with pentobarbital and transcardially perfused with 4% paraformaldehyde in 0.1 M PBS (pH 7.4). Brains were post-fixed overnight in 4% paraformaldehyde. Brains were dehydrated via 30-min incubations in a graded EtOH series (50%, 70%, 85%, 95%, and 100%) followed by tissue clearing with xylene. Brains were infiltrated with a graded paraffin series (30-min incubations of xylene:paraffin, 2:1, 1:1, 1:2) followed by two subsequent incubations in 100% paraffin for 1 hr. Paraffin-embedded brains were sectioned at 8 μm on a rotary microtome (Microm HM 360; Microm Laborgerate GmbH, Walldorf, Germany) and mounted onto Fisherbrand^™^ Superfrost^™^ Plus Microscope Slides (ThermoFisher Scientific; Waltham, MA, USA). Slides were baked at 60°C for 30 min, deparaffinized, and postfixed in 4% paraformaldehyde in 0.1M PBS for 15 min at 4°C. Sections were washed in 0.1 M diethyl pyrocarbonate (DEPC)-PBS, serially dehydrated with ethanol in DEPC-PBS (50%, 70%, and 100%), and then treated with H_2_O_2_ for 10 min. Sections were washed 3X with ultrapure water and transferred to 1X target retrieval reagent for 15 min at 99°C, followed by rinsing in ultrapure water. Sections were dehydrated again in 100% EtOH for 3 min and then dried for 5 min at 60°C. A hydrophobic barrier pen (Vector Laboratories; Newark, CA, USA) was used to draw barriers around tissue sections. Sections were pretreated with protease plus for 30 min. Sections were incubated with transcript-specific probes (Table S1) for 2 hr at 40°C using the HybEZ^™^ II Oven (Advanced Cell Diagnostics; Newark, CA, USA). Slides were washed 2X before the fluorescent multiplication assay was performed with hybridizing Amp 1-FL for 30 min at 40°C. After washing, we hybridized with Amp 2-FL for 15 min at 40°C. After washing, the next step was hybridization with Amp 3-FL for 30 min at 40°C. After washing, compatible fluorophores Opal^™^ 520, Opal^™^ 570, and Opal^™^ 620 were added to slides for 15 min each at 40°C. After washing slides, DAPI was added to slides and incubated for 30 s at room temperature. Slides were rinsed and blotted prior to the addition of mounting media and application of coverslips. Slides were stored in the dark at 4°C prior to imaging.

### RNAscope in situ hybridization (smISH)

We used the RNAscope^™^ 2.5 HD Assay-Brown (Table S1) workflow to generate permanent staining patterns of single mRNA molecules for the major and long *Gnal* isoforms. The overall protocol is like that presented for smFISH, but the brown detection kit has different steps for color development. The brown color detection assay was performed with hybridizing Amp 1 for 30 min, Amp 2 for 15 min, Amp 3 for 30 min, Amp 4 for 15 min, Amp 5 for 30 min, and Amp 6 for 15 min at 40°C. After each hybridization step, 1X wash buffer was used to wash sections 2X for 3 min each. DAB-A and DAB-B were mixed equally and added to the sections for 10 min to detect signals at room temperature. Gill’s hematoxylin was used to counterstain the slides for 10 min at RT. After dehydration, mounting media was applied to the slides to permanently fix them with coverslips.

### Imaging

Images were captured using a Nikon Ti-E A1Rsi confocal laser scanning inverted fluorescence microscope (Nikon Americas Inc., Melville, New York, USA). During image collection with a high resolution, the pinhole radius was set to 30.65 for all three channels. The Nikon NIS-Elements ND2 platform was used for image acquisition, processing, and analysis. QuPath^19^ was used to analyze the RNAscope smFISH signals. Plots were generated with GraphPad Software (Boston, MA, USA) using counts per region of interest (ROI). An Olympus BX63 microscope was used for bright field imaging of smISH. For qualitative analyses, an Akoya PhenoImage (Akoya Biosciences: Marlborough, MA, USA) was used for multispectral imaging of smFISH (DAPI [blue], Opal 520 [green, Gα(olf) transcripts], and Opal 570 [yellow, XLGα(olf) transcripts]).

### RNA-seq

A total of 16 samples (8 cerebellum, 8 striatum) were collected from four *Gnal*^+/−^ mice (two females and two males) and 4 *Gnal*^+/+^ littermates (two females and two males), all matched for age (13–17 months). Mice were deeply anesthetized with pentobarbital and then perfused with cold saline, followed by Invitrogen RNA*later* Stabilization Solution (Thermo Fisher Scientific, Waltham, MA, USA) to preserve RNA integrity. Samples were processed by GENEWIZ^®^ (Azenta Life Sciences, South Plainfield, NJ, USA) for RNA isolation, library preparation, and sequencing. The RNA library was prepared with high-quality total RNA, and mRNA was isolated with poly (A) selection. Then, cDNA synthesis, adaptor ligation, and PCR amplification were performed. High-throughput sequencing was performed with an Illumina^®^ NovaSeq^™^ 6000 to collect high-quality short-read cDNA, configured as 2 × 150 base pairs. High-throughput sequencing was conducted with single nucleotide resolution, with more than an 8,000-fold dynamic range for expression levels, and low background noise. Reads were mapped to the *Mus musculus* GRCm38 ERCC genome reference available on ENSEMBL, employing STAR aligner v.2.5.2b to align the complete read sequences, resulting in BAM files. Data quality was assessed with FastQC. The mean quality score for the eight samples was 37.46. On average 87.62% of bases had a quality score ≥ 30 (range 82.33 to 92.01%). The sequence reads were trimmed to eliminate adaptors and low-quality nucleotide sequences using fastp v.0.23.1. Unique gene hit counts were determined using featureCounts from the Subread package v.1.5.2, with the hit counts summarized and reported based on gene_id within the annotation file. Only unique reads that aligned to exon regions were counted. Using DESeq2, a comparison of gene expression between *Gnal*^+/−^ and *Gnal*^+/+^ samples was conducted, applying the Wald test to generate *p*-values and log2 fold changes.

### Transcriptomic bioinformatics

Bioinformatic analyses used the open-source programming language R (v 4.2.3). RNA-seq data derived from the cerebellae and striata of *Gnal*^+/−^ and *Gnal*^−/−^ adult mice were received from GENEWIZ with *p*-values, −log2FoldChanges, and false discovery rate (FDR) adjusted p-values (*adj_p*). Volcano plots, heatmaps, Top-level Gene Ontology (GO) analyses, and Gene Ontology (GO) analyses were generated by writing commands within RStudio. GO analyses used differentially expressed genes (DEGs) with *p* < 0.05. Cytoscape (cytoscape.org) embedded within Metascape was used to generate and visualize enrichment networks where nodes or enriched terms were grouped based on shared members thereby allowing identification of related processes. Metascape (metascape.org) tool was also used for gene annotation^20^.

### Human GNAL bioinformatics

These analyses focused on the deleteriousness and pathogenicity of variants affecting Exon 1 of NM_182978.4 (long isoform) and Exon 1 of NM_001369387.1 (major isoform) using gnomAD (gnomad.broadinstitute.org), ClinVar (www.ncbi.nlm.nih.gov/clinvar/) and UCSC Genome Browser (genome.ucsc.edu, GRCh38/hg38). These exons are unique to each isoform. Deleteriousness was assessed with CADD^21^ and REVEL^22^.

## Results

### Expression of Gα(olf) and XLGα(olf) transcripts in mouse brain

**Figure 1:**
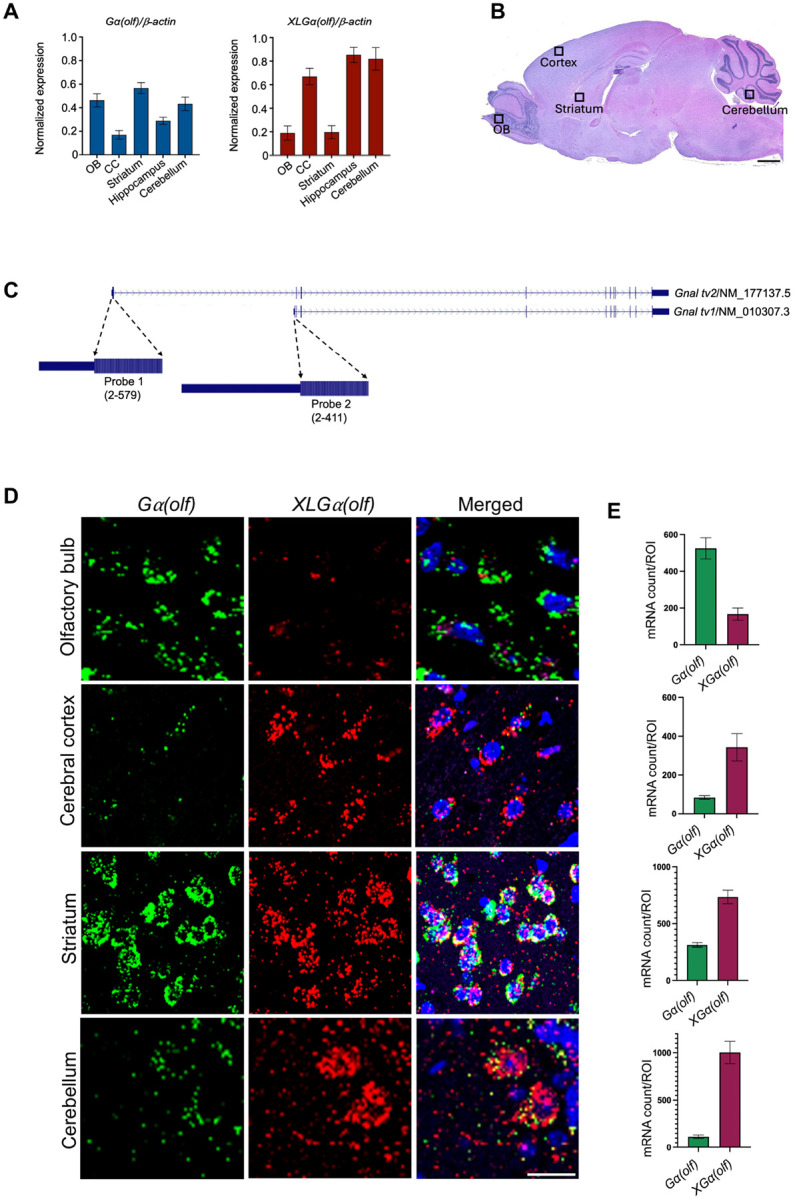
Expression of Gα(olf) and XLGα(olf) transcripts in adult mouse. **A**) Relative normalized expression of Gα(olf) and XLGα(olf) transcripts in adult mouse olfactory bulb [OB], cerebral cortex [CC], striatum, hippocampus, and cerebellum as found with RT-qPCR. **B**) Illustrative representation of areas selected for RT-qPCR and ISH in the adult mouse brain sagittal section [scale bar = 1 mm]. **C**) *Gnal* isoforms NM_177137.5 [XLGα(olf)] and NM_010307.3 [Gα(olf)] with the exonic nucleotide targets for locations of RNAscope^™^ probes. **D**) ISH localization of Gα(olf) (Opal 520, green) and XLGα(olf) transcripts (Opal 570, red) in the olfactory bulb [granule cell layer], cerebral cortex, striatum, and cerebellar cortex with DAPI nuclear counterstain with region of interest [ROI] quantification. Scale bar = 20 μm. **E**) Values are the mean ± standard error of the mean for 10 ROIs.

*Gnal* Gα(olf) and XLGα(olf) transcripts were widely expressed in mouse brain. The Gα(olf) transcript (NM_010307.3) was expressed at higher levels than the XLGα(olf) transcript (NM_177137.5) in the olfactory bulb and striatum [Fig. 1A – 1B]. In contrast, the XLGα(olf) transcript (NM_177137.5) was expressed at relatively higher levels in cerebral cortex, hippocampus and cerebellum [Fig. 1A – 1B]. For ISH, probe sets were specific to each isoform (Fig. 1C). More specifically, Exon 1 of NM_177137.5 is unique to NM_177137.5 and Exon 1 of NM_010307.3 is unique to NM_010307.3. In the granule cell layer of the olfactory bulb, Gα(olf) transcript was more abundant than XLGα(olf) transcript (Fig. 1D). There was modest cellular co-localization of Gα(olf) and XLGα(olf) transcripts in the granule cell layer olfactory bulb (Fig. S1 - S2). Overall, XLGα(olf) transcripts were more diffusely distributed in than Gα(olf) transcripts in the olfactory bulb. Both Gα(olf) and XLGα(olf) transcripts were concentrated in the mitral cell layer of the olfactory bulb (Fig. S3 – S4).

**Figure 2:**
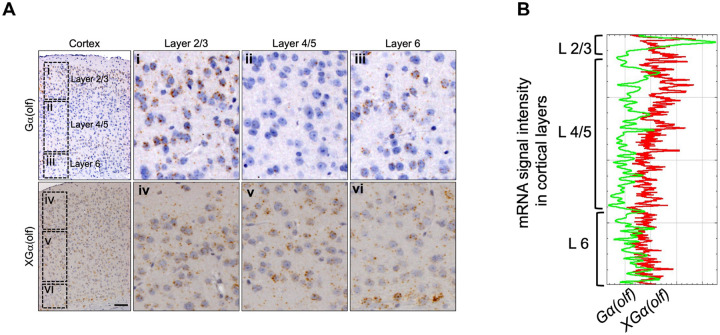
Expression of Gα(olf) and XLGα(olf) transcripts in the layers of adult mouse cerebral cortex. **A**) ISH images from cerebral cortex with cut-out enlargements for cortical layers 1–6. **B**) Plot profile quantifying transcript signal intensities throughout cortical layers. Scale bar = 50 μm.

In cerebral cortex, Gα(olf) transcripts were more sparsely distributed than XLGα(olf) transcripts (Fig. 1D). Gα(olf) transcripts were most prominent in Layers 2 and 3 while XLGα(olf) transcripts were abundantly present in layers 2–6 (Fig. 2A – 2B). XLGα(olf) transcripts were particularly dense in the lowest portions of Layer 6. In the stratum, both Gα(olf) and XLGα(olf) transcripts were present in most neurons (Fig. 1D, Fig. S5 - S6). Overall, however, Gα(olf) transcripts were moderately more abundant than XLGα(olf) transcripts.

**Figure 3:**
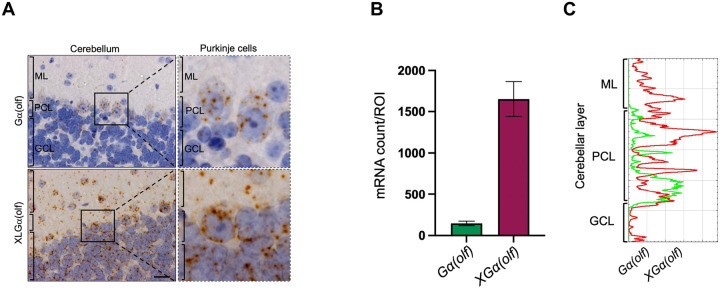
Expression of Gα(olf) and XLGα(olf) transcripts in the molecular (ML), Purkinje cell (PCL) and granule cell (GCL) layers of cerebellar cortex. **A**) ISH images show clear differences between the two *Gnal* isoforms. Scale bar = 20 μm. Image analysis with bar plots **B**) used 10 ROIs encompassing all cerebellar cortex layers. **C**) The plot profile is focused on the PCL and contiguous portions of the ML and GCL.

XLGα(olf) transcripts were abundantly expressed in all three layers (molecular, Purkinje cell, and granule cell) of cerebellar cortex while Gα(olf) transcripts were concentrated in the Purkinje cell layer (Fig. 1D and Fig. 3). Moreover, Gα(olf) transcripts appeared to be present in Purkinje cells rather than basket cells or contiguous glia (Fig. 3A).

### Differential gene expression in adult Gnal^+/−^ mouse cerebellum and striatum

In cerebellum, *Gnal* was down-regulated in *Gnal*^+/−^ mice (log2FoldChange = −0.56, *p* = 0.0013, *adj_p* = 0.72). Gnal down-regulation was also seen in the striatum (log2FoldChange = 0.82, *p* = 0.0049, *adj_p* = 0.89). These mean differences in down-regulation between the cerebellum and striatum were not statistically significant.

In striatum, a total of 161 genes showed differential expression with p < 0.01 (Supplementary Material). None of these genes survived correction for false discovery (*adj_p* > 0.05). Irrespective of *p* values, 2 genes were down-regulated with log2FoldChange < 2.0 and only 1 gene was up-regulated with log2FoldChange > 2.0. In cerebellum, a total of 168 genes showed differential expression with *p* < 0.01 (Supplementary Material). None of these genes survived correction for false discovery (*adj_p* > 0.05). Irrespective of p values, 22 genes were down-regulated with log2FoldChange < 2.0 and only two genes were up-regulated with log2FoldChange > 2.0.

**Figure 4:**
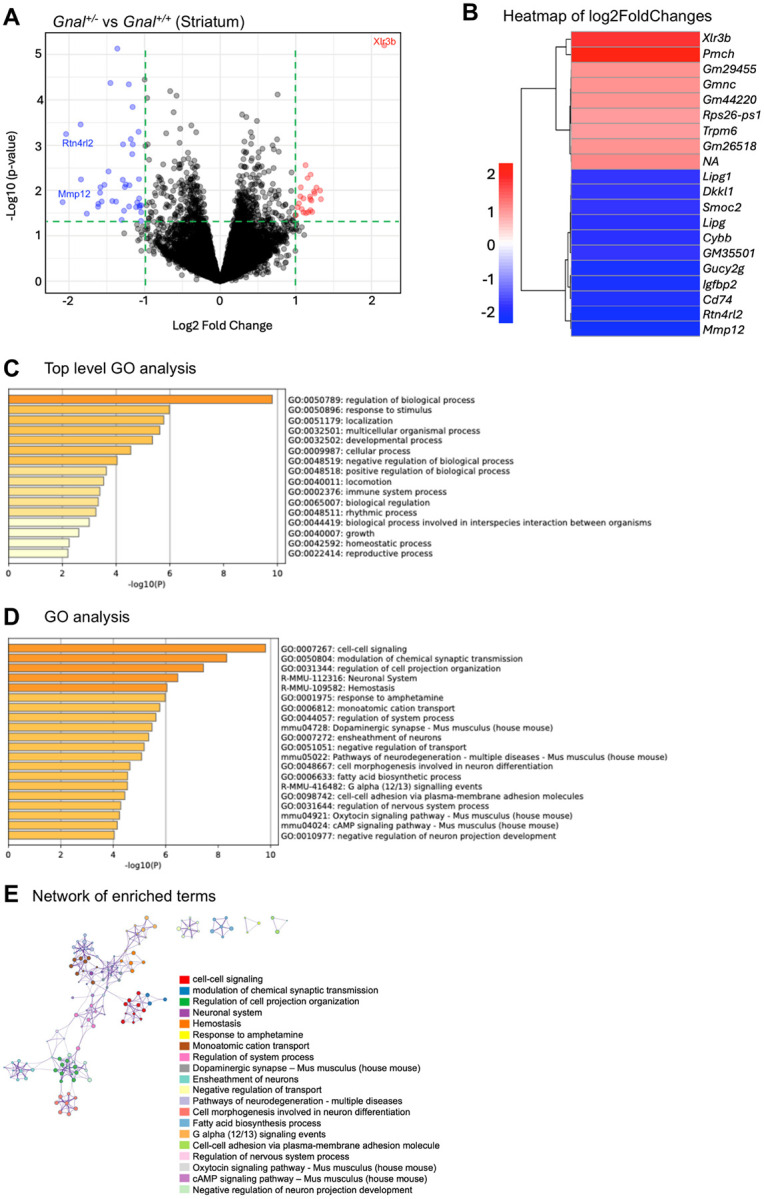
RNA-Seq analysis of *Gnal*^+/−^ mouse striatum. **A**) Volcano plot showing DEGs in *Gnal*^+/−^ versus wild-type (WT, *Gnal*^+/+^ littermates) adult mouse striatum. Blue dots stand for downregulated genes (most significantly *Rtn4rl2* and *Mmp12*), while red dots show upregulated genes (most significantly *Xlr3b*). **B**) Heatmap of DEGs after filtering based on the log2FoldChange. **C**) Top-level GO analysis for the biological processes of the identified DEGs in the striatum. **D**) Bar graph from GO analysis illustrates the top twenty enriched terms. **E**) A network of enriched terms colored by cluster ID based on the *p*-value of DEGs, where nodes sharing the same cluster ID are typically situated close to each other.

**Figure 5:**
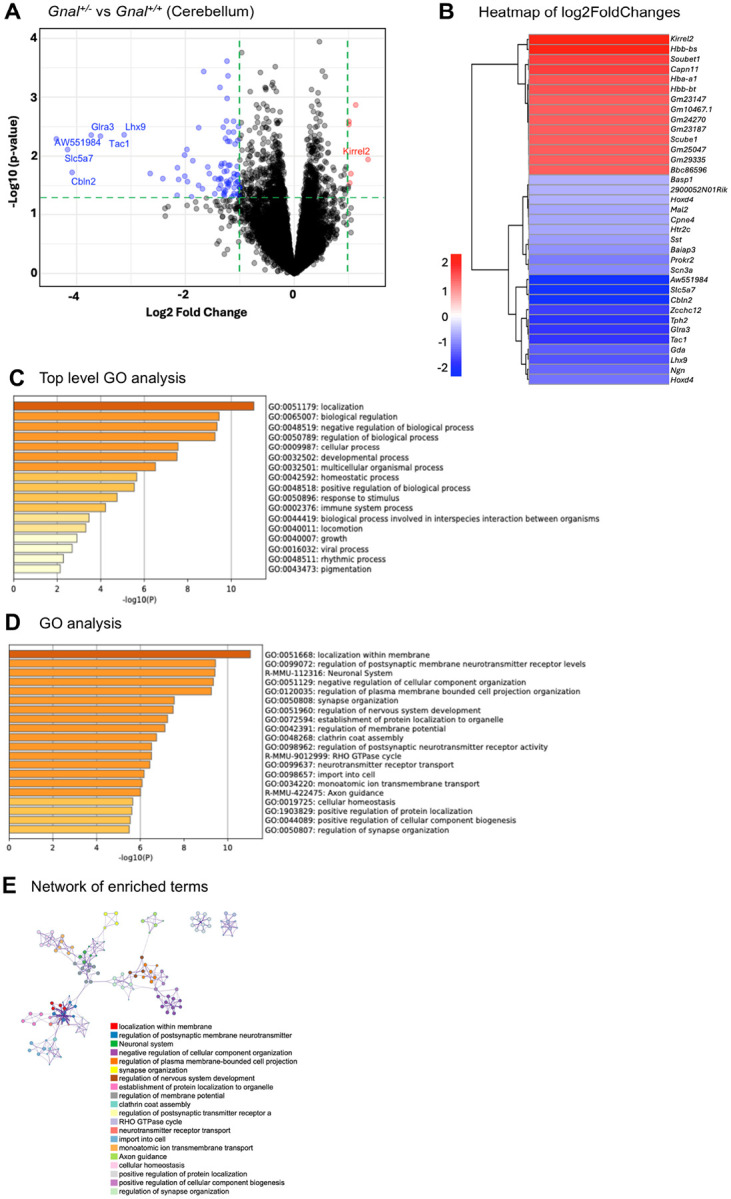
RNA-Seq analysis of *Gnal*^+/−^ cerebellum. **A**) The volcano plot displays DEGs for the cerebellum collected from *Gnal*^+/−^ versus WT in adult mouse brains. Blue dots show downregulated genes (most significantly *AW551984, Lhx9, Tac1, Cbln2, Glra3, Slc5a7*), while red dots stand for upregulated genes (unavailable). **B**) Heatmap showing the up- and down-regulated DEGs from the *Gnal*^+/−^ versus WT groups, filtered based on log2FoldChange. Color code values are described in the figure. **C**) Top-level GO analysis for the biological processes of the identified DEGs in the cerebellum. **D**) Bar graph from GO analysis illustrates the top twenty enriched terms. **E**) A network of enriched terms, colored by cluster ID based on *p*-values for DEGs, where nodes sharing the same cluster ID are typically positioned close to one another.

Volcano plots and heatmaps are shown in Fig. 4 (striatum) and Fig. 5 (cerebellum). In striatum, notably downregulated genes included *Mmp12* and *Rtn4rl2*. *Xlr3b* showed the largest magnitude of upregulation in striatum. In cerebellum, there were also a much larger number of genes showing downregulation than upregulation. Noteworthy were *Lhx9, Clbn2, Glra3*, and *Slc5a7*. *Kirrel2* showed the largest magnitude up-regulation in cerebellum.

In Table 1, we provide details on genes showing the largest log2FoldChanges. Several genes related to cell differentiation and development and DNA-templated transcription were down-regulated. None of the upregulated or downregulated genes list in Table 1 were common to the striatum and cerebellum. *Xlrb* showed the largest Log2FoldChange in the striatum and has been associated with cell differentiation, development, spine morphogenesis and synapse assembly. In striatum, several genes are associated with “immune system process.” GO Cellular Components included plasma membrane, nucleus, nuclear envelope, and extracellular region. GO Molecular Function included “transporter activity,” “DNA binding,” and “molecular transducer activity.”

Top-level GO analyses of the striatum (Fig. 4C) and cerebellum (Fig. 5C) showed important commonalities. GO terms negative regulation of biological process (0048519), positive regulation of biological process (0048519), immune system process (0002376), and developmental process (0032502) were common to both. In contrast, there were noteworthy differences in GO analyses between the striatum (Fig. 4D) and cerebellum (Fig. 5D). Cell-cell signaling (0007267) and modulation of chemical synaptic transmission (0050804) were the highest ranked GO terms in the striatum but neither appeared in the cerebellar GO list.

GO data does not show inter-cluster similarities or intra-cluster redundancies. To address this issue, we employed a visualization method for enrichment network analysis. Enrichment networks represent each node and the pairs of connecting nodes with kappa similarities > 0.3, forming a network that can be depicted using Cytoscape (cytoscape.org) embedded within Metascape. The size of each node represents its statistical *p*-value and cluster membership. Using DEGs lists to examine known protein interactions can expose signal transduction complexes that regulate cellular pathways. In Fig. 4E (striatum), note the close relationship between the “cell-cell signaling” and “modulation of chemical synaptic transmission” clusters. A large node related to “regulation of cell projection organization” is closely clustered with “negative regulation of neuron projection” and linked to a cluster related to “cell morphogenesis involved in neuron differentiation.” Different relationships were seen in the cerebellum (Fig. 5E). A “localization within membrane” node is closely clustered to “regulation of postsynaptic membrane neurotransmitter receptor levels” and “establishment of protein localization to organelle.”

### Human GNAL

Data from gnomAD_v4.1.0 and ClinVar (www.ncbi.nlm.nih.gov/clinvar/) was interrogated to gain insight on the relative contributions of the long and major isoforms of *GNAL* to dystonia and, human disease, in general (Tables 2 and 3). gnomAD_v4.1.0 includes data on 807,162 individuals (730,947 exomes and 76,215 genomes). gnomAD_v4.1.0 data was derived from over one hundred studies from over twenty-five countries. Over 17% of the individuals included in gnomAD_v4.1.0 harbored at least one defined phenotype such as Alzheimer’s disease, bipolar disorder, or inflammatory bowel disease. None of the studies were focused on dystonia and it appears unlikely that dystonia was exclusionary for most of the studies. Moreover, dystonia phenotypes may be subtle and often undiagnosed or misdiagnosed by non-neurologists. Analysis of gnomAD allows us to estimate the impact of variants at the population level. As seen in Table 2, the most important finding was the striking difference in the numbers of predicted loss of function (pLoF) variants between Exon 1 of the long and major isoforms of *GNAL*. At the most basic level, this strongly suggests that humans missing one copy of *GNAL* isoform NM_182978.4 can survive to adulthood without major neurological dysfunction. A total of 4 pathogenic pLoF variants (total allele count of 7/>1,586,000) were seen in Exons 2–12. Highly deleterious *GNAL* variants show reduced penetrance^5^ and it is likely that one or more of these individuals did not manifest dystonia.

ClinVar includes variants found through various sequencing platforms, both academic and commercial. Many disease-associated *GNAL* variants are not included in ClinVar. A PubMed search using the term *GNAL* did not find a co-segregating, highly deleterious NM_182978.4 Exon 1 variant is a multiplex pedigree with dystonia. A recent large scale whole-exome sequencing study did find a highly deleterious (CADD = 39.0) Exon 1 variant (GRCh38/hg38, NC_000018.10:g.11689900A > T,NM_182978.4:c.337A > T,p.Lys113*) in an individual with segmental dystonia^23^. Based on analysis of ClinVar data, the largest proportion of highly deleterious pathogenic variants are found within Exons 2–12 of both the major and long isoforms.

## Discussion

In this novel study, we detailed the localization of XLGα(olf) and Gα(olf) in mouse brain, documented the effects of XLGα(olf)/Gα(olf) haploinsufficiency on the mouse brain transcriptome, and examined the relative contributions of XLGα(olf) and Gα(olf) to dystonia and, in general, human disease. ISH showed that XLGα(olf) transcripts are more widely distributed in brain than Gα(olf) transcripts. Gα(olf) transcripts were present with more cell type specificity in cerebellar and cerebral cortex. Most neuronal populations expressed XLGα(olf) transcripts. The *Gnal*^+/−^ transcriptome exposed potential roles for XLGα(olf) and Gα(olf) in multiple cellular processes beyond the traditional conception of Gα(olf) as part of cell surface signaling. XLGα(olf) and Gα(olf) haploinsufficiency was associated with altered expression of genes related to transcription, developmental, immune system function, and differentiation. In human populations, heterozygous loss-of-function variants specific to XLGα(olf) appear to be better tolerated than those to Gα(olf). Highly deleterious variants specific to the major transcript of *GNAL* are uncommon in the human population. Our data suggests that both isoforms of *GNAL* are essential for normal neural function but the major isoform which encodes Gα(olf) may be more important in the pathobiology of dystonia.

We are not aware of validated antibodies specific to each isoform. In previous work, a rabbit polyclonal antibody was used to localize XLGα(olf)/Gα(olf)^5^ in rat brain. XLGα(olf)/Gα(olf)-immunoreactivity (IR) was detected in all major areas of neonatal and adult rat brain. In cerebellum, IR was most prominent in Purkinje cells. XLGα(olf)/Gα(olf)-IR was present throughout the striatum and substantia nigra pars compacta and reticulata and, in the striatum, co-localized with choline acetyltransferase (ChAT) in cholinergic neurons^5^. Olfactory expression of XLGα(olf)/Gα(olf) has been documented in several animal species including zebrafish^24^ and turtles. In mice, intense IR has been reported in the olfactory epithelium^18^. Overall, findings from immunohistochemistry are compatible with our findings using transcript specific ISH.

Prior ISH studies were not transcript specific^9,17,18^. ISH using digoxigenin-UTP and ^33^P-UTP-labeled probes localized XLGα(olf)/Gα(olf) to adult mouse caudate, putamen, nucleus accumbens, globus pallidus, substantia nigra, hippocampus, and cerebellar Purkinje cells^18^. More recent work with RNAScope^™^ localized XLGα(olf)/Gα(olf) transcripts to most neuronal groups in the mouse brain including cerebral cortex, striatum, hippocampus, substantia nigra and cerebellum^17^. Using cell-type specific probes, XLGα(olf)/Gα(olf) transcripts were specifically localized to medium spiny neurons of the direct and indirect pathways, dopaminergic neurons of the substantia nigra pars compacta, and cerebellar Purkinje cells^17^. Our findings are consistent with and expand upon earlier work and suggest that XLGα(olf) may play a more generalized role in neuronal functioning in contrast to the more circumscribed role (s) of Gα(olf) in cell-cell signaling.

Homozygous deletion of *Gnal* (*Gnal*^−/−^) is postnatally lethal in mice^18^. Fewer than 5% of homozygote pups survive for more than 30 days. Homozygous mice feed poorly, show hyperkinetic behavior, and are unable to breed. A homozygous *GNAL* mutation was associated with early onset generalized dystonia with mild intellectual disability in two Turkish siblings^25^. In both siblings, dystonia and cognitive dysfunction appeared after a period of normal development. The average age of onset for *GNAL*-associated dystonia is approximately 35 years of age (range 1 to 63 years)^4,5,26,27^. Only 10 to 15% of patients develop generalized dystonia. Some patients with *GNAL* mutations have microsmia^5^. The 18p deletion syndrome encompasses *GNAL* and is commonly associated with dystonia, intellectual disability, dysmorphism, and short stature^28,29^. Other genes within this locus include *TGIF1, SMCHD1, TUBB6, LAMA1, PIEZO2, MC2R, LPIN2, NDUFV2, AFG3L2* and *APCDD1*. Although the probable cause of dystonia in patients with the 18p deletion syndrome is haploinsufficiency of GNAL, it is possible that GNAL haploinsufficiency may contribute to other neural and extra-neural manifestations. In this regard, GNAL is widely expressed in extra-neural tissues, albeit at lower levels than in brain (Broad Institute, gtexportal.org) and may contribute to regulation of multiple biological processes (Figs. 4C and 5C).

G-protein coupled receptors regulate diverse cellular processes. Using lymphoblastoid RNA derived from four patients [Gα(olf), p.V228F] and four non-carriers from a single pedigree, we used the llumina^®^ HumanHT-12 v.4 expression microarray platform (Ilumina, San Diego, CA, USA) microarray platform to identify DEGs. Dysregulated networks in mutant lymphoblastoid cell lines were involved with cell cycle, development, cell death and cellular proliferation^5^. Although GNAL is expressed at low levels in lymphocytes, these findings show similarities to the results reported here from *Gnal*^+/−^ mouse brain.

XLGα(olf) and/or Gα(olf) interact with several nuclear proteins including USP3 (ubiquitin specific peptidase 3), BABAM1 (alias - MERIT40, BRISC and BRCA1 A complex member 1), and SPATA2 (spermatogenesis associated 2)^30–32^. Yeast-two hybrid screening followed by co-immunoprecipitation and focused knock-down experiments have been used to characterize the cellular functions of XLGα(olf)^15^. XLGα(olf) was shown to interact with CSN5 (COP9 signalosome subunit 5). CSN5 is part of the constitutive photomorphogenesis 9 (COP9) signalosome complex that plays a key role in cellular proliferation and senescence. CSN5 interacts with cyclin-dependent kinase 2 (CDK2)^33^. Silencing of CSN5 attenuated down-regulation of p27^Kip1^ encoded by *CDKN1B*. CDK2 participates in regulation of the cell cycle and DNA regulation while p27^Kip1^ can brake cell division by inhibiting CDK/cyclin complexes. These cellular studies are in line with those processes found to be dysregulated in *Gnal*^+/−^ mouse brain (Figs. 4 and 5), and our previous work showing that aged *Gnal*^+/−^ had increased DNA breaks, increased global DNA methylation, increased euchromatin, and dendritic structural abnormalities^16^. More work with mouse models along with human longitudinal neuroimaging and postmortem studies will be needed to determine if neurodegenerative processes contribute to the pathobiology of *GNAL*-associated dystonia.

Patterns of dysregulation in Gnal^+/−^ brain and specific DEGs are worthy of discussion. As seen from the volcano plots (Figs. 4A and 5A), a much larger number of genes showed downregulation than upregulation (> 1 log2FoldChange) and top-level GO analyses yielded the term “negative regulation of biological process” in both striatum and cerebellum. Of all genes examined, *Xlr3b* (encodes X-linked lymphocyte-regulated protein 3B) showed the largest degree of upregulation, perhaps to compensate for downregulation of several biological processes. XL3B participates in positive regulation of dendritic spine morphogenesis and synapse assembly^34^. In cerebellum, Kirrel2 was upregulated (Fig. 5A). KIRREL2 is a member of the neurexin family and contributes to synaptic development and neuronal adhesion particularly through cell-cell interactions. KIRRELS plays a critical role in cerebellar and olfactory development^35,36^. In cerebellum, *Cbln2* and *Lhx9* were downregulated (Fig. 5A). CBLN2 forms a complex (NRXN3-CBLN2-GLUD1) that downregulates postsynaptic AMPA-receptors^37^. LHX9, a DNA-binding transcription factor, has been linked to neuronal differentiation^38^. Reticulon-4 receptor like 2 (R4RL2 [Nogo-66 receptor homolog 1], *Rtn4rl2*) transcripts were downregulated in striatum (Fig. 4A, Table 1). R4RL2 is a cell surface receptor that regulates formation of dendritic spines and synapses during development^39^. Beyond the established role for Gα(olf) in medium spiny neuron cell-cell signaling, these results from RNA-Seq support roles for XLGα(olf) and/or Gα(olf) in neuronal differentiation and spine morphogenesis.

At the population level, pLoF variants solely affecting Exon 1 of the long *GNAL* isoform (NM_182978.4) are much more common than pLoF variants affecting Exon 1 of the major *GNAL* isoform (NM_001369387.1). In addition, pathogenic variants isolated to Exon 1 of the major *GNAL* isoform are more common than those isolated to Exon 1 of the long isoform. However, the large majority of published (PubMed) and reported (ClinVar) pathogenic dystonia-associated variants are in Exons 2 to 12 which are common to both isoforms. From a clinical standpoint, the keys to assessment of newly identified variants in patients with dystonia are details of the clinical phenotype^4,5,25,26^, deleteriousness scores (e.g., CADD and REVEL), co-segregation analysis when possible, and exclusion of other genetic causes. Most patients with *GNAL*-associated dystonia have onset in the neck with later spread to the face, masticatory muscles, and larynx, and some have clinically detectable hyposmia. Genetic diagnosis is important since patients may benefit from deep brain stimulation^40^.

The limitations of our work must be outlined. First, RNAScope^™^ localizes transcripts and not protein. In future studies, supplemental approaches such as isoform specific antibodies and tagging will be essential to study the relative roles of XLGα(olf) and Gα(olf) in neuronal biology. Second, transcript and protein levels are not linearly correlated. For instance, selective increases in Gα(olf) were observed in mice with dopamine D1 receptor and adenosine 2A receptor knock-out mice without changes in mRNA levels^8^. Furthermore, *Gnal*^+/−^ mice showed less than 50% reduction in transcript levels suggesting up-regulation of the intact allele. Isoform expression can be independent or co-regulated. This could affect interpretation of genetic databases. For example, deletion of the long isoform (NM_182978.4) could be well tolerated if the intact allele produced more transcript or intact transcripts produced more XLGα(olf). Future work should focus on the relative contributions of aberrant cell-cell signaling in the striatum and/or Purkinje cells and added cellular roles of XLGα(olf) and Gα(olf) including development, synapse formation, and differentiation to the pathobiology of dystonia.

## Supplementary Material

Supplementary Files

This is a list of supplementary files associated with this preprint. Click to download.

- SupplementaryFile.pdf
- SupplementaryInformationRNASeq.xlsx

## Figures and Tables

**Table 1:** Dysregulated genes in *Gnal*^+/−^ mice

Tissue	Gene	Log2 Fold Change	P value	GO Terms (Molecular Function)	GO Terms (Biological Process)	GO Terms (Cellular Component)
Cerebellum	*AW551984*	−4.37	0.005121974		cell differentiation, anatomical structure development	
	*Slc5a7*	−4.17	0.007736369	transporter activity	signaling, anatomical structure development, transmembrane transport	endosome, plasma membrane, cytoplasmic vesicle
	*Cbln2*	−4.087	0.018896845		signaling, cell junction organization, anatomical structure development	extracellular matrix, extracellular space
	*Glra3*	−3.737	0.004396193	transporter activity, molecular transduction activity	signaling, cell junction organization, nervous system process, transmembrane transport	plasma membrane
	*Tac1*	−3.567	0.00458412		inflammatory response, signaling, nervous system process	extracellular region
	*Lhx9*	−3.13	0.004349855	DNA binding, transcription regulator activity	regulation of DNA-templated transcription, reproductive process, cell differentiation, anatomical structure development	nucleus, chromosome
	*Col25a1*	−2.65	0.019745394	other	cell differentiation, anatomical structure development	extracellular region, extracellular space, plasma membrane, extracellular matrix
	*Cpne4*	−2.42	0.024118736	lipid binding, calcium-dependent phospholipid binding		glutamatergic synapse,
	*Htr2c*	−2.15	0.046300282	molecular transducer activity	signaling, cell differentiation, nervous system process, transmembrane transport	plasma membrane
	*Zcchc12*	−2.15	0.015741482	molecular adaptor activity, transcription regulator activity	regulation of DNA-templated transcription, signaling	nucleus, chromosome
	*Mal2*	−2.13	0.0249353			plasma membrane, cytoplasmic vesicle
	*Scn3a*	−2.01	0.009583325	transporter activity	transmembrane transport	plasma membrane
	*Basp1*	−2.01	0.02189947	DNA binding, molecular adaptor activity, transcription regulator activity	regulation of DNA-templated transcription, cell differentiation, anatomical structure development, protein-containing complex assembly	nucleus, nucleoplasmin, chromosome, plasma membrane
	*Gm24270*	1.13	0.001355547	lncRNA		
	*Kirrel2*	1.36	0.011411528		cell adhesion	plasma membrane
Striatum						
	*Mmp12*	−2.08	0.01825525	DNA binding, hydrolase activity, catalytic activity acting on a protein	immune system process, regulation of DNA-templated transcription, intracellular protein transport, nucleocytoplasmic transport, cell adhesion, signaling, protein catabolic process, wound healing, anatomical structure development, cell motility	extracellular region, nucleus, extracellular matrix
	*Rtn4rl2*	−2.04	0.000565771	molecular transducer activity	signaling, cell differentiation, anatomical structure development	plasma membrane
	*Gucy2g*	−1.85	0.000347721	cyclase activity, transferase activity, lyase activity, catalytic activity	signaling, nucleobase-containing small molecule metabolic process, carbohydrate derivative metabolic process	plasma membrane
	*Igf2bp2*	−1.84	0.005774448	RNA binding, molecular adapter activity	mRNA metabolic process	nucleus, cytoskeleton
	*Cd74*	−1.76	0.032701522	protein folding chaperone, molecular transducer activity	immune system process, regulation of DNA-templated transcription, protein folding, lipid metabolic process, intracellular protein transport, cell adhesion, programmed cell death, signaling, cell differentiation, anatomical structure development,	extracellular region, nucleus, lysosome, endosome, endoplasmic reticulum, Golgi apparatus, plasma membrane
	*Cybb*	−1.61	0.023021772	oxidoreductase activity	immune system process, inflammatory response, signaling, anatomical structure development, transmembrane transport	nuclear envelope, mitochondrion, endoplasmic reticulum, plasma membrane
	*Lpg*	−1.61	0.019966366	hydrolase activity	lipid metabolic process	extracellular space, endosome, Golgi apparatus,
	*Dkkl1*	−1.59	0.011345998		lipid metabolic process, programmed cell death, reproductive process, signaling, cell differentiation	extracellular region, cytoplasmic vesicle
	*Iigp1*	−1.58	0.0086196	cytoskeletal motor activity, GTPase activity	immune system process, autophagy, signaling, defense response to other organism	extracellular region, nuclear envelope, endoplasmic reticulum, Golgi apparatus
	*Smoc2*	−1.57	0.017718378		cell adhesion, extracellular matrix organization	extracellular region, extracellular matrix
	*Gm35501*	−1.53	0.007613271	lncRNA		
	*Gm44220*	1.27	0.008781636	lncRNA		
	*Gm26518*	1.32	0.01012378	lncRNA		
	*Xlr3b*	2.17	6.33E-06		cell differentiation, cell junction organization, anatomical structure development, positive regulation of dendritic spine morphogenesis, positive regulation of synapse assembly	

**Table 2:** Comparison of exon 1 long and major isoforms (gnomAD_v4.1.0)

	Exon 1 (NM_182978.4)	Exon 1 (NM_001369387.1)
	Long Isoform	Major Isoform
Nucleotide length	676	494
Total number of variants	620	358
Mean CADD score	14.61	10.80
Number of missense variants	247	36
ClinVar number of variants within gnomAD	27	8
ClinVar Benign	7	1
ClinVar Likely Benign	5	5
ClinVar Uncertain significance	15	2
Mean CADD score ClinVar variants	15.64	16.48
pLoF (predicted Loss of Function) variants	37	1

**Table 3:** ClinVar variants

	Exon 1 (NM_182978.4)	Exon 1 (NM_001369387.1)	Exons 2 – 12
	Long Isoform	Major Isoform	
# missense variants	15	6	50
# missense variants associated with dystonia	8	5	24
CADD scores (mean, range)	17.0 (0.26 – 35)	20.4 (15.9 – 22.1)	29 (18.1 – 34)
REVEL_rankscores (mean, range)	0.54, 0.18 – 0.99	0.49, 0.30 – 0.86	0.89, 0.19 – 0.94
#frameshift and nonsense variants	0	3	8

## Data Availability

The datasets used and generated during the current study are available from the corresponding author upon request.
